# Longitudinal in-vivo quantification of tumour microvascular heterogeneity by optical coherence angiography in pre-clinical radiation therapy

**DOI:** 10.1038/s41598-022-09625-8

**Published:** 2022-04-12

**Authors:** Nader Allam, W. Jeffrey Zabel, Valentin Demidov, Blake Jones, Costel Flueraru, Edward Taylor, I. Alex Vitkin

**Affiliations:** 1 Department of Medical Biophysics, University of Toronto, 101 College Street, Toronto, ON M5G 1L7 Canada; 2 Geisel School of Medicine at Dartmouth, 1 Rope Ferry Rd, Hanover, NH 03755 USA; 3 National Research Council Canada, Information Communication Technology, 1200 Montreal Rd, Ottawa, ON K1A 0R6 Canada; 4 Radiation Medicine Program, Princess Margaret Cancer Centre, 610 University Avenue, Toronto, ON M5G 2M9 Canada; 5 Department of Radiation Oncology, University of Toronto, 149 College Street, Toronto, ON M5T 1P5 Canada

**Keywords:** Cancer models, Interference microscopy, Predictive markers, Cancer imaging, Tumour biomarkers, Tumour heterogeneity

## Abstract

Stereotactic body radiotherapy (SBRT) is an emerging cancer treatment due to its logistical and potential therapeutic benefits as compared to conventional radiotherapy. However, its mechanism of action is yet to be fully understood, likely involving the ablation of tumour microvasculature by higher doses per fraction used in SBRT. In this study, we hypothesized that longitudinal imaging and quantification of the vascular architecture may elucidate the relationship between the microvasculature and tumour response kinetics. Pancreatic human tumour xenografts were thus irradiated with single doses of $10$, $20$ and $30$ Gy to simulate the first fraction of a SBRT protocol. Tumour microvascular changes were monitored with optical coherence angiography for up to $8$ weeks following irradiation. The temporal kinetics of two microvascular architectural metrics were studied as a function of time and dose: the diffusion-limited fraction, representing poorly vascularized tissue $>150$ μm from the nearest detected vessel, and the vascular distribution convexity index, a measure of vessel aggregation at short distances. These biological metrics allowed for dose dependent temporal evaluation of tissue (re)vascularization and vessel aggregation after radiotherapy, showing promise for determining the SBRT dose–response relationship.

## Introduction

Recent technological advances in image-guided radiotherapy have opened the door to the delivery of hypofractionated ablative radiotherapy (RT), most notably stereotactic body radiotherapy (SBRT), by ensuring that doses to organs-at-risk remain acceptable^1–3^. SBRT has shown promise for improving local tumour control for many of the most lethal cancer types such as pancreatic ductal adenocarcinoma^4–6^. It remains an open question, however, whether its apparent enhanced efficacy is due only to its higher biologically effective doses, or whether an alternate mechanism of action is at play, over and above the standard linear quadratic model of tumour cell kill through the DNA damage mechanisms^7–10^.

It has been suggested that high doses per fraction ($>8-10$ Gy) ablate small blood vessels (diameter $\lesssim 30$ μm)^11–13^ which in turn leads to an improved tumour response by depriving it of nutrients necessary to regrow post-SBRT^14,15^. The most important such nutrient is oxygen, due to its role in the fixation of RT-induced DNA damage^16^ and subsequent tumour metabolism and re-growth^17^. Indeed, the change in oxygen levels throughout the course of fractionated radiotherapy—a process known as “reoxygenation”—is one of the major predictors of tumour response^18^. The role of microvasculature may prove even more important in SBRT, with its higher-doses-per-fraction regimen appearing to invoke additional effects beyond the cellular DNA damage mechanisms^11–13^. Thus, the temporal kinetics of the microvascular response to SBRT are likely a major determinant of its efficacy and need to be studied in detail.

Towards this goal, we have developed an optical coherence tomography angiography (OCA) platform to measure and quantify the temporal kinetics of the vascular volume density (VVD), defined as the total proportion of the tumour volume occupied by the vasculature, in response to high single-dose irradiation in a pre-clinical in-vivo study^15^. OCA is well-suited for this longitudinal investigation due to its capacity to resolve the microvascular structures which appear to be most affected by SBRT’s higher radiation doses^15,19,20^ as well as enabling high-contrast, *in-vivo* angiography, label-free and non-invasively in 3D up to a depth of $\sim 1-2$^ mm15,21,22^. That work established vascular regrowth via angiogenesis as an important precursor of tumour relapse, suggesting that the VVD metric is a potentially useful vascular biomarker for gross tumour response to high dose irradiation. However, it is also an oversimplification, in that the ability of a tumour vasculature to deliver nutrients, to influence RT efficacy, and to affect re-growth kinetics is unlikely to be captured in a single vessel density number. Indeed, the well-known high spatial heterogeneity of the tumour microenvironment^23–25^ is not reflected in the VVD metric that averages microscopic morphological changes of the microvascular network, and more informative quantifiers should be derived. Hence, this work sought to address the need for new OCA-derived metrics that may be: (1) more sensitive to microvascular heterogeneity and (2) capable of quantifying changes in the efficiency of transport of nutrients/drugs/oxygen from the vasculature to the tissue.

The temporal kinetics of two such potential metrics for describing the vascular geometry captured via an OCA technique called speckle variance optical coherence tomography (svOCT), following high single-dose irradiation, are investigated here: the diffusion limited fraction ($DL{F}_{\Lambda }$), and the vascular convexity index ($\lambda$)^26^. The feasibility of using svOCT to measure these metrics longitudinally in time, in response to RT of different doses, is part of our larger research effort towards “shedding light on radiotherapy”.

The former biomarker is defined as the fractional volume of tissue beyond a given distance $\Lambda$ from the nearest blood vessel. For compounds such as oxygen or chemotherapy drugs that are metabolized or otherwise taken up in cells, the molecules will diffuse a characteristic distance $\sim 2\sqrt{D/M}$ from the blood vessels, before being metabolized^27^. Here, $D$ and $M$ are the diffusivity and rates of metabolism/uptake respectively. For oxygen, using $D = 2000$ μm^2^/s and $M = 0.375$ s^-1 ^(corresponding to a consumption rate of $15$ mmHg/s and an assumed capillary oxygen partial pressure of $40$ mmHg), Thomlinson and Gray estimated this distance to be $\sim 150$^ μm28^. Thus, $DL{F}_{\Lambda }$ with $\Lambda =150$ μm ($DL{F}_{150}$) characterizes the fraction of tissue in a tumour for which oxygen may be low, impacting the efficacy of fractionated RT and the ability of tumour cells to proliferate^29^. A two-dimensional analogue of this metric, the diffusion limited (area) fraction DLAF, was proposed by Janssen and colleagues for resected tumour tissue slices, by analyzing the spatial distribution of histological blood vessel markers^25^. However, because DLAF ignores the 3D volumetric nature of the microvascular network and must be quantified *ex-vivo* (from invasive biopsy which may also suffer from preparation artefacts), histopathological analysis may not be an optimal tool to study the longitudinal evolution of this quantity, further motivating the significance of our svOCT-based *in-vivo* 3D approach.

While the $DL{F}_{150}$ describes the presence of significantly large “avascular” voids, the convexity index $\lambda$ was proposed by Baish et al*.* to quantify the shape of the distance-to-nearest-vessel histogram at “short” distances ($\lesssim 50-100$ μm, approximately equal to the average inter-vessel distance)^26^. It can be viewed as a metric for quantifying the efficiency with which a given tissue vasculature can deliver nutrients to proliferative cancer cells nearest to blood vessels.

## Materials and methods

### Ethics approvement

All animal handling procedures were approved by the University Health Network’s institutional Animal Care Committee in accordance with the guidelines of the Canadian Council for Animal Care (animal use protocol #3256). Proper measures were taken to minimize animal discomfort through administration of anesthesia and analgesia as necessary. Results of this study are reported in compliance with the ARRIVE guidelines^30^.

### Tumour xenograft model & experimental treatment

NOD-Rag1^null^ IL2rg^null^ (NRG) mice were selected as the animal model for this longitudinal study due to their radioresistance comparable to humans and high immunodeficiency suitable for tumour generation^31^. All animal procedures and data acquisition protocols have been described previously^15^. In brief, 2.5 × 10^5^ human BxPC-3 cells, labeled with Discosoma sp. Red Orange Mushroom (DsRed) derived red fluorescent proteins (Anticancer Inc., San Diego, CA, USA) and suspended in $10$ μL of 1:1 PBS:Matrigel (BD, Biosciences, ON, Canada) were injected subcutaneously and dorsally in NRG mice ($n=18$). Once tumours reached a diameter of $3-5$ mm, titanium dorsal skin window chambers (DSWC) were installed to enable longitudinal intravital optical monitoring in response to a range of single fraction irradiations: $10$ Gy ($n=5$), $20$ Gy($n=4$), $30$ Gy ($n=6$), and unirradiated controls ($n=3$). Mice were randomly assigned to these treatment groups with full knowledge from all investigators^30^. Tumours were treated using an X-RAD 225Cx small animal irradiator (Precision X-ray, North Branford, CT) through an $8$ mm diameter collimator, at $225$ kVp and $13$ mA, with a $0.3$ mm Cu filter, corresponding to a dose rate of $2.63$ Gy/min at the tumour^32^. The system was calibrated following the American Association of Physicists in Medicine TG-61 protocol^33^.

### Tumour response monitoring

Tumour macroscopic and microvascular monitoring was performed every $2-4$ days prior- and post-irradiation for up to $2$ months. Gross-tumour response to irradiation was assessed longitudinally through two independent metrics: tumour volume (via caliper measurements) and tumour viability (via quantification of the average intensity of red fluorescent protein expression at $535$ nm excitation, $580$ nm emission wavelengths fluorescence imaging (Leica Microsystems, Richmond Hill, Canada)) (data previously presented^15^).

Volumetric microstructural imaging of the tumours was performed at $8\times 15\times 15$ μm^3 ^(${axial} \times {lateral}^{2}$) resolution within a $6\times 6$ mm^2^ field-of-view (FOV) up to $\sim 1$ mm in depth via a swept-source OCT system operating at $1320$ nm center wavelength with $110$ nm bandwidth, and an A-scan rate of 20 kHz. Eight B-scans were sequentially recorded ($\sim 25$ ms apart in time) per lateral step ($\sim 15$ μm apart)^15^. This allowed for contrast enhancement of blood vessels with respect to surrounding static tissue and hence microangiography, via speckle-variance (sv) processing:

1 $$sv\left[I\left(z,x\right)\right] =\sum_{t=1}^{M}\frac{{\left(\overline{I\left(z,x\right)}-{I}_{t}\left(z,x\right)\right)}^{2}}{M},$$

where $I(z,x)$ denotes the OCT signal intensity at coordinates z pixels along the depth and x pixels along the width of the captured B-mode frame in each lateral step, $\overline{I\left(z,x\right)}$ is the average signal intensity across the $M$ sequentially acquired same-position B-scans (here $M=8$); see ref.^21,22^ for more details.

### Tissue & vascular segmentation

**Figure 1:**
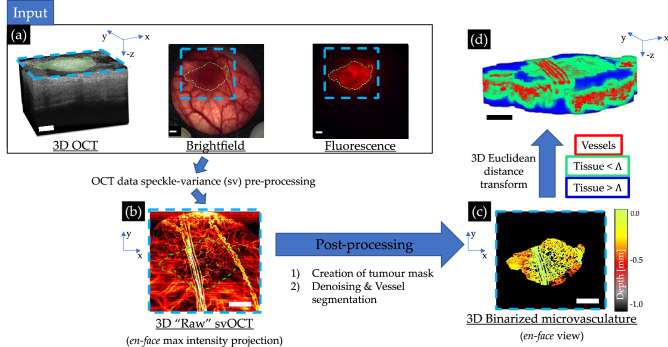
svOCT microvascular segmentation pipeline to enable extraction of diffusion limited fraction $DL{F}_{\Lambda }$ and the vascular convexity index $\lambda$: (**a**) 3D structural OCT image along with corresponding brightfield and DsRed fluorescence micrographs; blue contours indicate the FOV of the corresponding OCT scan, green contours the tumour in the lateral plane; (**b**) *en-face* 2D maximum intensity projection of the resultant microvasculature obtained via svOCT processing using Eq. (1); (**c**) depth-encoded *en-face* projection of the binarized microvasculature in the segmented tumour VOI; (**d**) Colour-coded 3D representation of distance transformed binarized svOCT volume delineating vessels (red), as well as both proximal ($\lesssim 150$ μm, green) and distant ($\gtrsim 150$ μm, blue) tissue voxels from the nearest vessel. This final image enables all computations pertaining to the distance to nearest vessel (DNV) histograms (for details, see text, Sect.  Tissue microvasculature heterogeneity quantification). Scale bars are $1$ mm, 3D volumes are $6\times 6\times 1$ mm^3 ^(${lateral}^{2}\times depth$). All images above are derived from the same mouse-timepoint data acquisition. Green lateral tumour contours were generated based on the raw brightfield and fluorescence images (shown above) using MATLAB R2021a software. OCT-derived images were generated by processing the same raw OCT acquisition using custom scripts in MATLAB R2021a software (https://github.com/nallam1/Batch-processing-multimodality-tumour-imaging). Organization of the images for display was completed in Microsoft PowerPoint.

The obtained “raw” svOCT 3D microvascular maps were then segmented to prepare for metric extraction using a lab-designed pipeline implemented through MATLAB R2021a software (MathWorks, MA, USA) as represented in Fig. 1. To reduce speckle “salt & pepper” noise as well as motion artifacts during imaging, a series of morphological opening/closing operations and median filtering steps were applied^22^. To account for signal depth attenuation described by Beer-Lambert law, as well as to remove “shadowing” artifacts due to Mie scattering and absorption from red blood cells, a depth-decaying thresholding and a step-down exponential filter steps were applied^22^.

Tumour volume of interest (VOI) contours were delineated at each timepoint, using multi-modal acquisitions—brightfield microscopy, structural OCT, speckle-variance OCT, Ds-Red fluorescence—to reduce subjectivity and improve inter-timepoint VOI accuracy. Specifically, in the transverse (lateral) plane, a 2D tumour mask was created based on the fluorescence or brightfield images co-registered to a maximum intensity *en-face* projection of the subsequently acquired “raw” svOCT microvasculature images, benefitting from the enhanced normal tissue to tumour contrast afforded by these modalities. Due to accumulation of optically translucid exudate, arising from elevated cytokine activity in the DSWC over the period of weeks following surgery^34^ (seen in some of the mice), accurate manual contouring of the tissue interface along the axial (depth) direction via structural OCT was not feasible at all timepoints. Instead, the VOIs were contoured axially from the inner surface of the DSWC glass down to a fixed depth of $\sim 1$ mm, based on the 3D structural OCT scans. To the extent that the distance between the DSWC glass and tissue surface remained constant over time, this ensured a consistent VOI definition inter-timepoint. A downside of this approach is that small air gaps which may arise between the tissue and inner glass surface are not excluded from our analysis. Although these could modify the distance-to-nearest-vessel DNV histograms slightly (masquerading as ‘avascular tissue’ regions), especially at large distances, they would not appreciably affect the relative changes / kinetics of the histogram and related metrics, $DL{F}_{\Lambda }$ and $\lambda$.

All vessels within the delineated tumour VOI were then segmented with a Frangi-vesselness filter based on Hessian eigenvalue decomposition^35,36^. Finally, the generated 3D microvasculature map was binarized and visually compared to the “raw” svOCT volume to guide in removal of small artifacts. The binarized 3D vascular maps were rescaled to have isotropic voxel size of ($\sim 2.5$μm)^3^﻿ in order to compute the distance-to-nearest-vessel (DNV) histogram ${n}({\delta})$ with reduced binning artifacts^26^.

### Tissue microvasculature heterogeneity quantification

The diffusion-limited distance ${DLF}_{\Lambda }$ was calculated as the proportion of tissue voxels beyond a distance threshold $\Lambda \ge 150$ μm from the nearest vessel within the tumour VOI:

2 $${DLF}_{\Lambda }\equiv \frac{Tissue \, volume \, \left(\delta >\Lambda \right)}{Total \, tissue \, volume},$$

where $\delta$ is the distance of a given voxel of tissue to the nearest vessel. In terms of the unit-normalized DNV histogram ${n}({\delta })$ ($\sum_{\delta }{n}({\delta})=1$), it is

3 $${DLF}_{\Lambda }=\sum_{\delta \ge \Lambda }{n}({\delta}),$$

The convexity index $\uplambda$ is derived from the power-series fit to the log–log histogram of ${n}({\delta })$ versus $\delta$, assuming power-law behaviour at short distances^26^:

4 $${n}({\delta }) \sim {\delta }^{\lambda },$$

**Figure 2:**
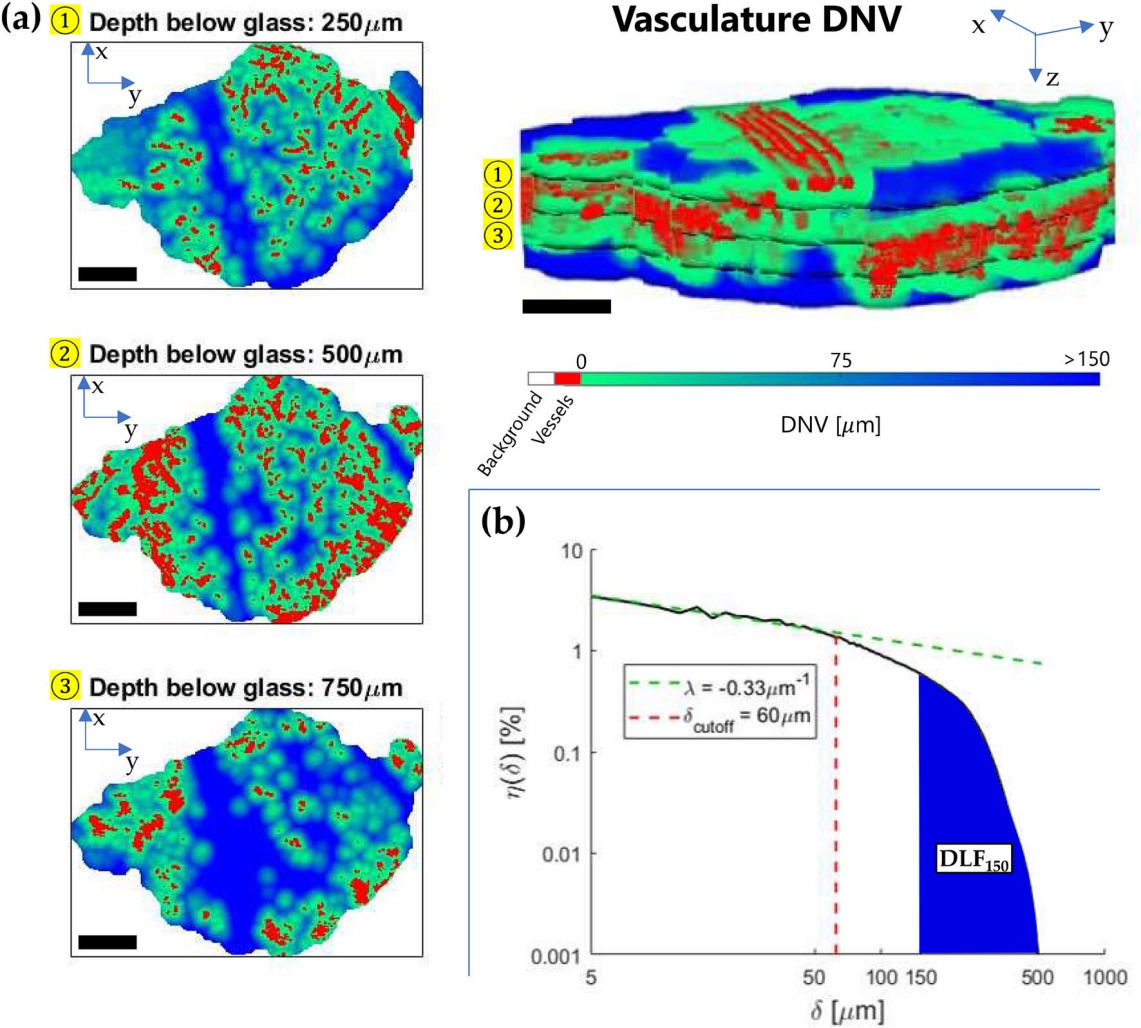
Evaluation of the diffusion-limited fraction $DL{F}_{150}$ and convexity index λ: (**a**) 2D visualizations of the colour-coded distance-to-nearest-vessel (DNV) map at three different depths below the window chamber to tissue interface, from the application of the 3D Euclidean distance transform to the binarized microvasculature within the tumour VOI. The upper right of the figure shows the full 3D vasculature and tissue distances parametric volumetric image; (**b**) The corresponding log–log DNV histogram for the full tumour VOI. The indicated $\lambda$ and $DL{F}_{150}$ metrics quantify short and long-distance properties of the DNV, respectively. Scale bars are $1$ mm, 3D volumes are  $6\times 6\times 1$ mm^3^ (${lateral}^{2}\times depth$). Each distance bin was $2.5$ μm, the rescaled isotropic voxel size in the 3D parametric images for smooth computations.

Baish et al.^26^ fit the DNV histogram using Eq. (4) for distances up to $\delta ={\delta }_{max}/3$, where ${\delta }_{max}$ is the maximum measured value of $\delta$. We used a fixed cutoff of $60$ μm based on the mean and median DNV which was also empirically found to most consistently quantify the short-distance range best modeled by a power-law. Illustrative $DL{F}_{150}$ and *λ* determinations are shown diagrammatically in Fig. 2.

### Statistical analysis

Vascular metrics $DL{F}_{150}$ and $\lambda$ data were acquired pre- and post-irradiation (generally less than one hour prior to and following irradiation) and subsequently every $2-4$ days for up to 2 months. All inter-treatment comparisons were performed using one-way analysis of variance (ANOVA) followed by two-sample t-test using Microsoft Excel. No data were excluded from the analysis. For all tests, p $<0.05$ was assumed significant.

## Results & discussion

**Figure 3:**
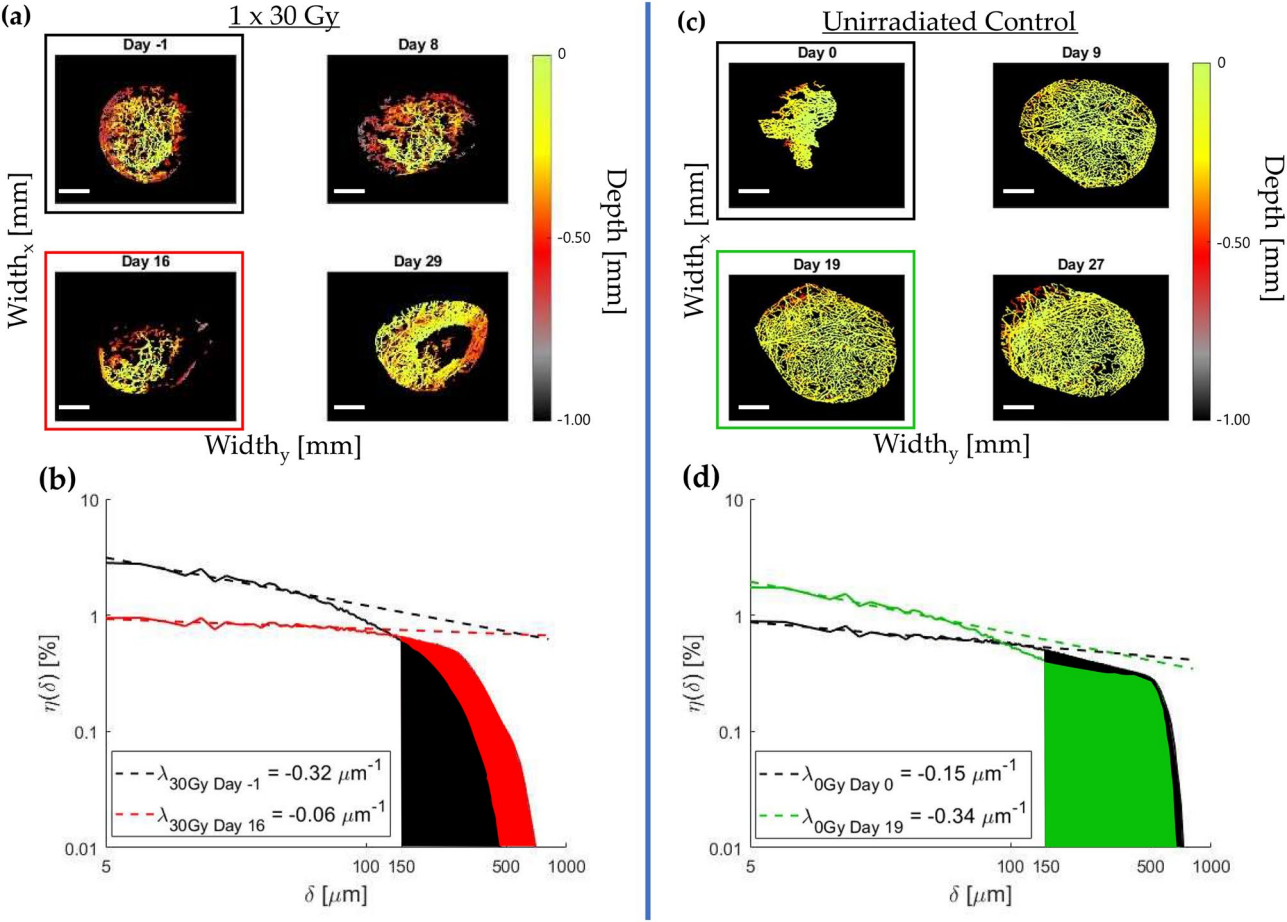
Illustrative changes in vascular architectures for irradiated and un-irradiated tumours, with corresponding $DL{F}_{150}$ and $\uplambda$ quantification: (**a**) $30$ Gy irradiated and (**c**) unirradiated control colour-depth encoded binarized vasculature within the tumour VOI for several timepoints over the course of a month; (**b**) and (**d**) show the corresponding $t \sim 0$ and $t \sim 2-3$ weeks DNV histograms for both mice, and the two derived metrics. The slope of the histogram at short distances (defining the convexity index $\uplambda$) generally increased after irradiation at this time (b), while the opposite was seen in unirradiated controls (d). For “avascular” regions as quantified by $DL{F}_{150}$, the trends were a general initial increase in $DL{F}_{150}$ with dose (b), while it slightly decreased in the absence of irradiation (d). More comprehensive time-dose summary for all animals is presented in Fig. 4. Scale bars are $1$ mm.

Representative temporal evolution of the 3D vasculature in the presence and absence of radiation, and quantification via DNV histogram analysis are shown in Fig. 3. As seen in the images (a) and (c), the microvascular architecture is highly variable with time, and its temporal dynamics are altered by radiation (as would be expected in this case for the rather large dose of $30$ Gy). Quantification of these rich and complex volumetric patterns is challenging, and panels (b) and (d) show the corresponding DNV histogram analysis to enable such quantification. The derived $DL{F}_{150}$ and $\lambda$ metrics for these two mice, shown at two representative times of the longitudinal imaging study, allow some quantification but also underscore the intra- and inter-animal variability that makes quantitative analysis challenging. We now carry out similar comprehensive analysis at all time points for all animals in the three dose cohorts (plus unirradiated controls) to reveal the overarching trends.

**Figure 4:**
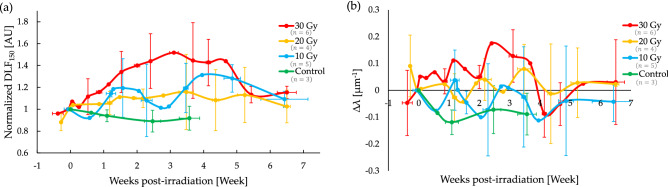
Temporal trends in $DL{F}_{150}$ and $\lambda$ of tumour vascular networks differ as a function of radiation dose: (**a**) Normalized $DL{F}_{150}$, plotted here as the ratio $DL{F}_{150}(t)/DL{F}_{150}(0)$, with $DL{F}_{150}(0)$ being the pre-irradiation value; (**b**) the change $\Delta \lambda (t)\equiv \lambda (t)-\lambda (0)$ in the convexity index relative to its pre-irradiation value, $\lambda (0)$. Initially, both $DL{F}_{150}$ and $\lambda$ increase before returning to baseline levels at $\sim 5-7$ weeks post irradiation; the convexity index behaviour seems “noisier” with significant temporal fluctuations. For further trends elucidation, see text. Symbols are experimental points and lines are a guide for the eye; error bars represent standard deviations amongst the different treatment cohorts.

Figure 4 displays the quantified results of the entire study. Both $DL{F}_{150}$ and $\lambda$ were found to change in time by an amount related to the dose levels before nominally returning to baseline levels. This return to “normal” pre-irradiation levels is not surprising as single dose treatments are essentially never used in the clinic. Nevertheless, investigating this logistically simpler single-dose treatment case prior to standard multi-fractionated SBRT is an important preliminary step for two primary reasons. (1) It eliminates sources of uncertainty pertaining to variability in inter-fraction tumour response dynamics and precision of administered fractions, thus optimizing the experimental protocol and data processing. (2) Investigating the effects of a single dose irradiation (with comparable equivalent dose, EQD, to a several weeks $2$ Gy fractions regimen) relative to a typical SBRT treatment improves our understanding of the first-order effects of the SBRT treatment in general, and of its first-delivered fraction in particular.

Taking a closer look at the results summary of Fig. 4, we observe the following:

- Immediately following and for $\sim 2-3$ weeks after irradiation, both $DL{F}_{150}$ and $\lambda$ exhibited an increase; the trends in the convexity index are harder to elucidate due to considerable temporal λ-fluctuations. Overall magnitudes of increase seem to scale with dose.
- $DL{F}_{150}$ and $\lambda$ both attained their maximum values between $2-3$ weeks post-irradiation. Compared to unirradiated controls, $DL{F}_{150}(2.5\,\,{ \text{weeks}})/DL{F}_{150}(0)$ was significantly larger after $30$ Gy ($p = 0.0021$) and $20$ Gy $(p = 0.015$) but not after $10$ Gy $(p = 0.13$). Similarly, relative to controls, $\Delta\uplambda$($2.5 \,\,{\text{weeks}}$) was significantly larger for tumours treated with $30$ Gy ($p = 0.0041$), but not with $20$ Gy ($p = 0.31$) nor with $10$ Gy ($p =0.53$).
- After $\sim 3$ weeks post-irradiation, both $DL{F}_{150}$ and $\lambda$ gradually returned to their pre-irradiation levels.
- Unirradiated controls exhibited a slight decrease over time for both metrics.

Previous work has established that the vascular volume density (VVD) decreases post-irradiation in a dose-dependent manner using the same tumour model as explored here^15^. It was further hypothesized that the VVD kinetics could predict tumour volume growth dynamics, based on modelling work by Kozin and collaborators^14,15^. In the current study, we have examined the feasibility of measuring additional architectural vascular metrics longitudinally in time, hypothesizing that these may reveal more about tumour functionality and ultimately allow for better tumour dose–response prediction compared to VVD alone.

The diffusion-limited fraction $DL{F}_{150}$ is the fraction of tissue that is more than $150$ μm from the nearest blood vessel and thus represents tissue regions which may be deprived of nutrients, in particular oxygen^25,28^. Hypoxic cancer cells are known to be non-proliferative, potentially more metastasizing, and resistant to radiation^29,37^. Consequently, an evolving $DL{F}_{150}$ during radiotherapy may impact tumour growth in several ways. First, an increasing $DL{F}_{150}$ means that a greater proportion of tissue may be more resistant to radiation, via the oxygen enhancement ratio effect^18,29,38^. Second, this same fraction of tissue may become non-proliferative, enhancing the impact of radiation on tumour volume growth delay^14,29^.

The relationship between $DL{F}_{150}$ and hypoxia is not clear-cut, however, since hypoxia depends not only on vessel architecture but also perfusion—the flow rate of oxygen-carrying blood through vessels—as well as tumour metabolism. While there is clear evidence for an oxygen gradient that scales with distance-from-nearest-blood-vessels (“chronic hypoxia”)^24,25,39^, inter- and intra-subject heterogeneity in perfusion and metabolism spatio-temporally^23^ challenge the identification of a universal relationship between hypoxia and $DL{F}_{150}$^25^.

Given that large doses of radiation ablate blood vessels, it is arguably not surprising that $DL{F}_{150}$ increased during the $2-3$ weeks following irradiation, before decreasing again as new vessels were formed; analogous temporal changes were observed with the VVD metric examined earlier^15^. However, the precise relationship between $DL{F}_{150}$ and VVD encodes potentially important architectural information suggesting that there is additional new insights described by the former. For instance, for a randomly arranged array of parallel cylinders of uniform radii ${r}_{c}$, $DL{F}_{150}$ follows a Poisson distribution:

5 $${ln DLF}_{150} \sim {-\left(\frac{150}{{r}_{c}}\right)}^{2}\cdot \mathrm{VVD}$$

**Figure 5:**
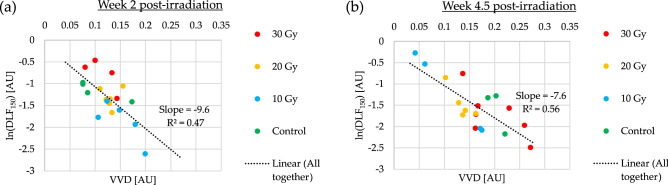
The diffusion-limited fraction $DL{F}_{150}$ versus vascular volume density VVD metrics at two timepoints post irradiation. (**a**) $t = 2$ weeks. (**b**) $t = 4.5$ weeks. The change in this relationship over time suggests an evolving microvascular architecture response to radiation.

Deviations from this relationship thus describe the inhomogeneous structure in the vascular architecture, beyond simple randomness. In Fig. 5, we plot the logarithm of $DL{F}_{150}$ versus VVD at two timepoints post-irradiation for all examined tumours. The temporal change in the relationship between these two quantities supports our hypothesis that $DL{F}_{150}$ contains architectural information that is distinct from, and perhaps complementary to VVD.

The convexity index $\lambda$ describes the rate of change of the distance-to-nearest-vessel DNV histogram $n\left(\delta \right)$ at “short” distances $\delta$ (on the order of the mean DNV, $\overline{\delta}$ and shorter). Normal tissue histograms are characterized by an $n\left(\delta \right)$ that increases with increasing $\delta$ ($\lambda >0)$, reaching a maximum close to the mean value, $\overline{\delta}$, before receding to ~ zero at long distances^26^. A positive value of $\lambda$ represents a vasculature that efficiently distributes nutrients to tissue, insofar as fewer blood vessels are needed for $\delta <\overline{\delta}$; e.g., if oxygen emanating from a given blood vessel can diffuse $\sim 150$ μm before being metabolized, there is no need to have an abundance of blood vessels that are $\lesssim 50-100$ μm  from each other. In contrast to the typical normal tissues, tumour vasculatures are often highly irregular, with a near monotonically decreasing $n\left(\delta \right)$, meaning that chemotherapy drugs and oxygen are inefficiently delivered by the vasculature^26,40^.

The fact that $\lambda$ generally increased after irradiation for larger doses over the first ~ 3 weeks is thus noteworthy (Fig. 4). It implies that radiation may have preferentially ablated vessels that were densely packed together, perhaps “normalizing” the vasculature in a manner analogous to that proposed as a therapeutic strategy using anti-angiogenic agents^40^. This may be a useful finding for locally advanced pancreatic cancers, where chemotherapy is the backbone of treatment to mitigate metastatic dissemination and questions surrounding the best way to combine radiotherapy with chemotherapy exist^41^. It remains to investigate whether this effect persists for more clinically relevant, fractionated radiation schedules.

The temporal fluctuations in $\lambda$, essentially not observed in $DL{F}_{150}$, likely arise from the high sensitivity of this curve-fitting based metric to the short distance range where we expect most speckle artefacts missed during the post-processing (see Sect. Tissue & vascular segmentation). With improved automation of both tumour^42^ and especially vessel segmentation^43^, the accuracy and inter-timepoint consistency of the OCA post-processing should be significantly increased. Applying these improvements along with a larger sample size to mitigate the influence of biological variability and possible post-processing related uncertainties, the observed temporal fluctuations in the $\lambda$ metric should be greatly reduced. However, noting the trends’ potentially regular undulations (~ one week period), it is also possible that radiobiological mechanisms may be involved pertaining to the complex interplay between pro- and anti- angiogenic factors^14,15,44–46^. Future studies should elucidate this behaviour.

While fluctuations in $10$ and  $20$Gy cohorts seemed to reduce statistical significance of trends relative to control, there is nevertheless an underlying trend of the vascular metrics scaling with dose. This is supported by the statistical significance of our $30$ Gy cohort results and the literature^14,15,47,48^.

**Figure 6:**
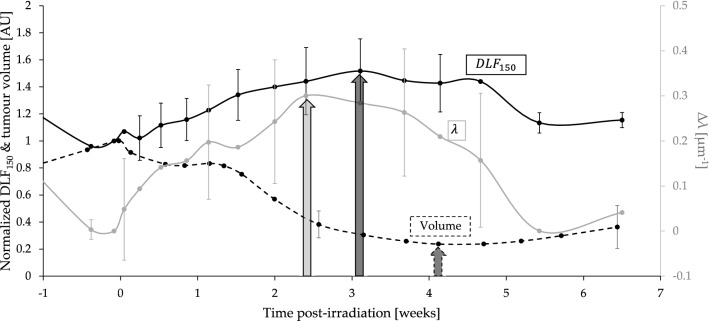
$DL{F}_{150}$ & $\lambda$ metrics show consistent and earlier trends compared with the tumour volume response, showing promise as candidate predictive biomarkers. The mean normalized $DL{F}_{150}$ (solid black, left y-axis) and λ (solid grey, right y-axis) for the $30$ Gy treated mice $(n=6)$ seem to precede the temporal evolution of the mean normalized tumour volume (dotted black, left y-axis). Arrows represent approximate time-to-peak in response maxima and minima, respectively. Error bars represent standard deviations.

It is interesting to note as well that despite the modest sample size of this study, both metrics showed trends which *preceded* that of the macroscopic tumour tissue response as seen in see Fig. 6. This further suggests their potential clinical value as candidate predictive biomarkers of tumour response during and following treatment. Although both $DL{F}_{150}$ and $\lambda$ changed significantly during RT, both quantities returned to their baseline values after $5-6$ weeks post single-fraction RT, a result of blood vessel re-growth^14^. Consequently, there may be a narrow window of time in which vessel architectural changes can have an impact on RT response.

## Conclusion

This study presents microvasculature assessment of pancreatic human tumour xenografts irradiated with $10$, $20$and $30$Gy doses, and OCT angiography performed for up to 8 weeks following irradiation. We further elucidate the temporal kinetics of tumour microvasculature response to high dose radiation regimes such as in SBRT, specifically accounting for the important factor of microvascular heterogeneity, which to our knowledge has not been previously investigated longitudinally *in-vivo*. Therefore, the temporal trends in the vascular distribution convexity index ($\lambda$) metrics and the diffusion-limited fraction ($DL{F}_{150}$) were extracted and presented per dose cohort. As measures of the short- and long-distance scales of the vascular distribution respectively, they not only contribute to a more complete description of microvascular heterogeneity but may also correlate with clinically relevant metric including hypoxic fraction and vascular transport efficiency. These biological metrics thus allowed for dose-dependent accurate identification of tissue revascularization and vessel aggregation dynamics after radiotherapy, showing their promising use in the determination of the SBRT dose–response relationship. Future work will focus on testing their performance in other tumour models and using clinically relevant SBRT fractionation schedules (e.g., $3\times10$ Gy every other day).

## Data Availability

Data underlying the results presented in this paper may be obtained from the authors upon reasonable request.
